# Safety and Feasibility of Vulvar Cancer Treatment with Electrochemotherapy

**DOI:** 10.3390/cancers15123079

**Published:** 2023-06-07

**Authors:** Gregor Vivod, Masa Bosnjak, Nina Kovacevic, Gregor Sersa, Sebastjan Merlo, Maja Cemazar

**Affiliations:** 1 Department of Gynecological Oncology, Institute of Oncology Ljubljana, 1000 Ljubljana, Slovenia; gvivod@onko-i.si (G.V.); nkovacevic@onko-i.si (N.K.); 2 Faculty of Medicine, University of Ljubljana, 1000 Ljubljana, Slovenia; 3 Department of Experimental Oncology, Institute of Oncology Ljubljana, 1000 Ljubljana, Slovenia; mbosnjak@onko-i.si (M.B.); gsersa@onko-i.si (G.S.); 4 Faculty of Pharmacy, University of Ljubljana, 1000 Ljubljana, Slovenia; 5 Faculty of Health Care Angela Boskin, 4270 Jesenice, Slovenia; 6 Faculty of Health Sciences, University of Ljubljana, 1000 Ljubljana, Slovenia; 7 Faculty of Medicine, University of Maribor, 2000 Maribor, Slovenia; 8 Faculty of Health Sciences, University of Primorska, 6000 Izola, Slovenia

**Keywords:** electroporation, vulvar cancer, safety, feasibility, electrochemotherapy, bleomycin

## Abstract

**Simple Summary:**

A prospective institutional study was conducted at the Institute of Oncology in Ljubljana. The main objective of the study is to determine the safety and feasibility of electrochemotherapy in the treatment of vulvar cancer recurrence. From July 2020 to January 2023, 10 patients with vulvar cancer recurrence were enrolled in our study. Patients were treated with an intravenous application of bleomycin, and 8 min later, electric pulses were applied locally to the tumor to increase the cytotoxicity of bleomycin. The treatment could be performed in all patients, demonstrating its feasibility, and no adverse effects were documented, proving that the treatment is safe. In conclusion, this is the first prospective study of electrochemotherapy in the treatment of local vulvar cancer recurrence conducted for nonpalliative purposes, demonstrating its safety and feasibility.

**Abstract:**

Electrochemotherapy is a local ablative therapy used for the treatment of various superficial and deep-seated tumors. Electrochemotherapy involves the application of electric pulses locally to tumors to destabilize cell membranes and facilitate the entry of cytotoxic drugs, thereby enhancing their cytotoxicity locally. The aim of our study is to investigate the safety and feasibility of electrochemotherapy in patients with vulvar cancer recurrence used for nonpalliative purposes. Ten patients with single local vulvar cancer recurrence were treated with intravenous bleomycin, followed by a local application of electric pulses (electrochemotherapy) to the tumor. Adverse events were determined using the National Cancer Institute’s Common Terminology Criteria for Adverse Events (CTCAE) version 5.0. The feasibility of treating vulvar cancer with electrochemotherapy was determined by an appropriate selection of electrodes based on the size and location of the tumor with safety margins included. Electrochemotherapy was feasible in all patients. No electrochemotherapy-related or other serious adverse events occurred. Our data suggest that electrochemotherapy is a feasible and safe technique for the treatment of vulvar cancer recurrence for nonpalliative purposes. Based on our results, electrochemotherapy might be a viable therapeutic tool for patients who would otherwise undergo surgery involving a mutilation of the external genitalia.

## 1. Introduction

Vulvar cancer accounts for 5–8% of all gynecologic cancers. Women over 65 years of age are most commonly affected [1]. Worldwide, approximately 45,000 cases of vulvar cancer are diagnosed each year, and 50.1% of patients are from highly developed countries in the Western world. A total of 17,000 women die from vulvar cancer each year [2]. Recently, the incidence of vulvar cancer has increased in the younger population, which has been attributed to HPV infection [3]. Squamous cell carcinoma accounts for 90% of all cases, followed by melanoma, adenocarcinoma, basal cell carcinoma, sarcoma, and the undifferentiated type. When vulvar cancer is diagnosed, patients may have no symptoms or may complain of problems, such as itching, a burning sensation, difficulty urinating, bloody discharge, and pain [4]. Vulvar cancer may present as thickened skin or as a flat, ulcerated lesion, or a wart-like formation above the skin level [1]. The appearance of vulvar cancer may be skin-colored, pale, red, or darkly pigmented [4]. The labia majora are the most common site of origin of vulvar cancer, accounting for approximately 50% of cases. The labia minora accounts for 15 to 20% of cases of vulvar cancer. The clitoris and Bartholin’s glands are less commonly affected [5].

Surgery and radiotherapy are the main treatment modalities in the management of vulvar cancer, at present. The final decision of vulvar cancer treatment depends on the staging, age of the patient, performance status, and presence of comorbidities [2]. The status of the tumor margin is considered an important prognostic factor for vulvar cancer recurrence, and radical local excision with a margin of at least 1–2 cm is recommended [1]. To achieve a free tumor surgical margin status, the surgery should often be radical and include a partial resection of the urethra, anus, and vagina. Consequently, radical surgery in the vulvar area may lead to urinary and fecal incontinence, scarring, and the mutilation of the external genitalia. Surgical morbidity also includes infection, wound dehiscence, and urinary- and gastrointestinal-related complications [6]. Vulvar cancer recurs in 37% of patients within five years. Most recurrences (40–80%) occur within the first two years after initial treatment. Treatment is often difficult and requires an individualized approach, depending on the site of recurrence and prior treatment [2]. Deciding how to treat a recurrence is often challenging. In recent years, the approach to treating disease recurrence has changed toward less invasive treatment. Therefore, treatment should be individualized and performed in specialized cancer centers with a multidisciplinary team [7].

Electrochemotherapy is a local ablative therapy used for superficial tumors, such as squamous cell carcinoma, basal cell carcinoma, melanoma, sarcoma, and skin metastases from breast cancer [8]. It is also used for deep-seated tumors, such as hepatocellular carcinoma or liver metastases from colon cancer [9]. In electrochemotherapy, electric pulses are applied to tumors through specific electrodes with different geometries, which are designed to cover the entire tumor and have an adequate safety margin with the electric field [10]. This leads to destabilization of the cell membranes, facilitating the diffusion of cytotoxic drugs into the tumors and potentiating local cytotoxicity. Two drugs are most commonly used for electrochemotherapy: bleomycin and cisplatin. The route of drug administration is either an intratumoural injection for both bleomycin and cisplatin or an intravenous bolus infusion when bleomycin is used. The decision for administration is based on the number and size of the tumors; for larger and multiple tumors, intravenous administration is recommended [11]. This therapy is conducted following standard operating procedures, and it is used in nearly 180 cancer centers around the world, to date [11]. Electrochemotherapy has also been listed as a treatment option in several national guidelines and for the treatment of metastatic melanomas in the ESMO guidelines [12]. A recent analysis of the International Network for Sharing Practice on Electrochemotherapy (InspECT) database, including 987 patients with 2482 tumor lesions of different histologies, showed an overall response rate of 85%, with 70% complete and 15% partial response rates [8]. For squamous cell carcinomas, the complete and partial response rates were 63% and 17%, respectively. In addition to being safe and effective as a form of monotherapy, electrochemotherapy can be successfully combined with other standard treatments, such as targeted therapies (vemurafenib and dabrafenib) [13] or immunotherapy (pembrolizumab) [14]. To date, only a few articles or clinical cases have been published describing the use of electrochemotherapy in patients with vulvar cancer for palliative purposes [15,16,17,18,19,20,21,22]. In these articles, the safety and local efficacy of electrochemotherapy have been demonstrated; however, a detailed analysis of the safety and feasibility of electrochemotherapy in vulvar cancer patients is lacking. In studies where electrochemotherapy was used with palliative intent for recurrent vulvar cancer, a high overall response rate of approximately 75–80% was achieved [15,16,17,18]. In addition, vulvar cancer recurrences treated with electrochemotherapy for palliative purposes were mostly up to 50 mm in size. It is known that the overall response rate for smaller cutaneous lesions is significantly better than the overall response rate for larger cutaneous lesions of more than 50 mm. For smaller lesions (less than 30 mm), the overall response rate is over 90% [8].

To the best of our knowledge, little is known about the potential complications of electrochemotherapy in patients with vulvar cancer, especially with regard to the risk of urethral or bowel injuries. Considering the increasing incidence of vulvar cancer mentioned earlier, especially in the younger population, electrochemotherapy might be a treatment option in cases where patients would otherwise undergo surgery with the mutilation of their external genitalia. Therefore, the aim of our study is to evaluate the safety and feasibility of electrochemotherapy in patients with vulvar cancer recurrence.

## 2. Materials and Methods

### 2.1. Study Design

A prospective, institution-based study was conducted at the Institute of Oncology Ljubljana. Regulatory approval for the study was obtained from the Institutional Medical Board (number ERID–KSOPKR–0042/2021), as well as from the Slovenian National Ethics Committee (number 0120–262/2021/3). Signed informed consent was obtained from all patients included in the study. Patients were presented at interinstitutional tumor board meetings consisting of a gynecologic oncologist, a radiologist, a medical oncologist, a radiation oncologist, and a pathologist. Electrochemotherapy was performed according to standard operating procedures [11]. The main objective of the study was to determine the safety and feasibility of electrochemotherapy in the treatment of vulvar cancer recurrence for nonpalliative purposes.

### 2.2. Patients

From July 2020 to January 2023, 10 patients with vulvar cancer recurrence were enrolled in our study, based on the inclusion and exclusion criteria presented in Table 1. All included patients had single local vulvar cancer recurrence confirmed by an experienced pathologist. Regional and distant metastases were excluded by appropriate imaging.

In our study, we analyzed the clinical characteristics of the patients with treatment history and treatment data with details of the procedure and hospital stay.

### 2.3. Treatment Procedure and Feasibility Assessment

Electrochemotherapy with bleomycin was performed according to the standard operating procedure for electrochemotherapy [11]. Briefly, the procedure was performed under general anesthesia. Bleomycin was administered intravenously at a dose of 10,000 or 15,000 IU/m^2^ (Bleomycin Medac, Medac GmbH, Wedel, Germany), depending on the patient’s age and concomitant diseases. A lower dose was used for elderly, fragile patients with concomitant diseases. Eight minutes after the intravenous administration of the drug, electric pulses were applied to the tumors via specially designed electrodes in such a way that the entire tumor nodule was covered, including a safety margin of 1 cm.

The choice of electrodes was based on the size, location, and accessibility of the tumor nodule. For larger tumors, easily accessible needle electrodes with a hexagonal geometry were used. In the case of smaller tumors, it was more difficult to access finger electrodes with 6 needles arranged in 2 rows. Electric pulses were generated by the Cliniporator VITAE (IGEA S.p.A., Carpi, Italy). For the hexagonal electrodes, 96 pulses were applied with a voltage of 730 V, a pulse duration of 100 µs, and a frequency of 5 kHz, whereas for the needle electrodes, 8 pulses were applied with a voltage of 4000 V, a pulse duration of 100 µs, and a frequency of 5 kHz. The feasibility of vulvar cancer treatment with electrochemotherapy was defined by (i) the enrollment of patients, (ii) electrochemotherapy procedure, and (iii) treatment-response evaluation.

The reason for the decision to use electrochemotherapy was that, after the treatment, the slow resolution of the treated tumor followed. The underlying mechanism was apoptotic cell death when the cells underwent cell division. Therefore, cell death occurred due to the cytotoxicity of the drug, i.e., bleomycin, and did not die due to electroporation, as is the case in irreversible electroporation.

### 2.4. Safety Assessment

Adverse events after electrochemotherapy were determined using the National Cancer Institute Common Terminology Criteria for Adverse Events (CTCAE) version 5.0.

## 3. Results

Between July 2020 and January 2023, 10 patients were eligible for the study. The median age of patients was 79.6 years old (range 64–91) at the time of enrollment in the study. The median body mass index of patients was 24.96, and most patients were not overweight. Eight patients were evaluated as having a WHO performance status of 0 or 1, and two patients were evaluated as having a WHO performance status of 2. All patients were diagnosed with recurrent squamous cell vulvar carcinomas. Six patients had been previously admitted to surgery alone and four patients were previously treated with surgery and radiotherapy. Electrochemotherapy was offered to all patients to avoid extensive and mutilating surgery after previous surgeries, and because of the proximity or involvement of the urethra, clitoris, and perineum. The detailed clinical characteristics of the patients are shown in Table 2.

**Figure 1:**
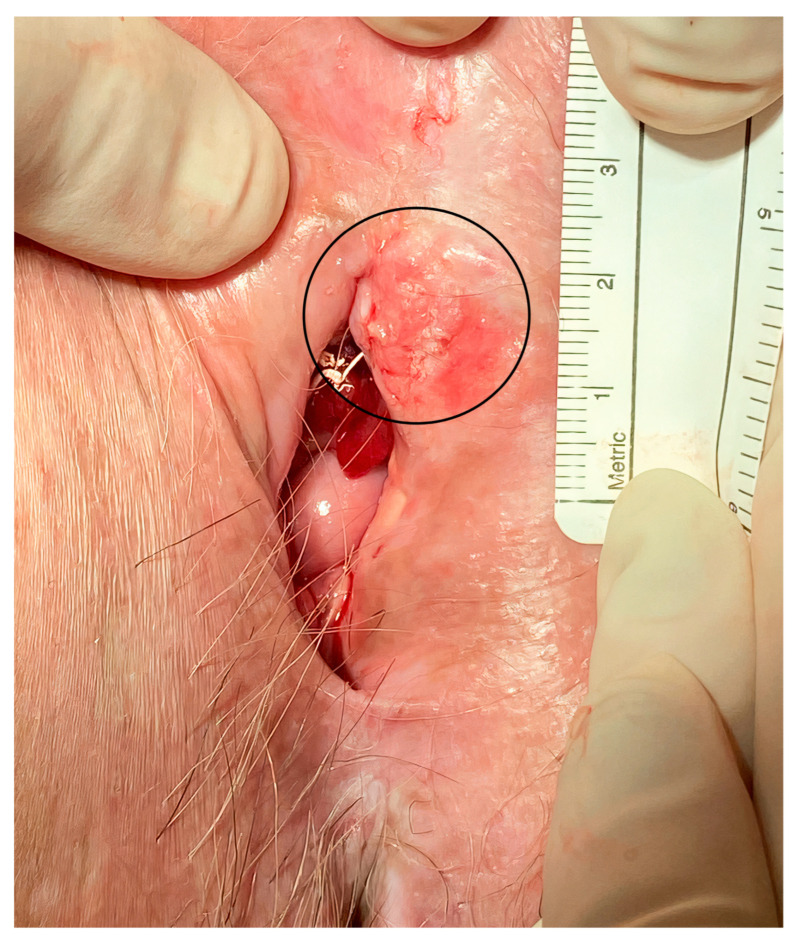
Local recurrence of vulvar cancer (circled line) in the periurethral area.

**Figure 2:**
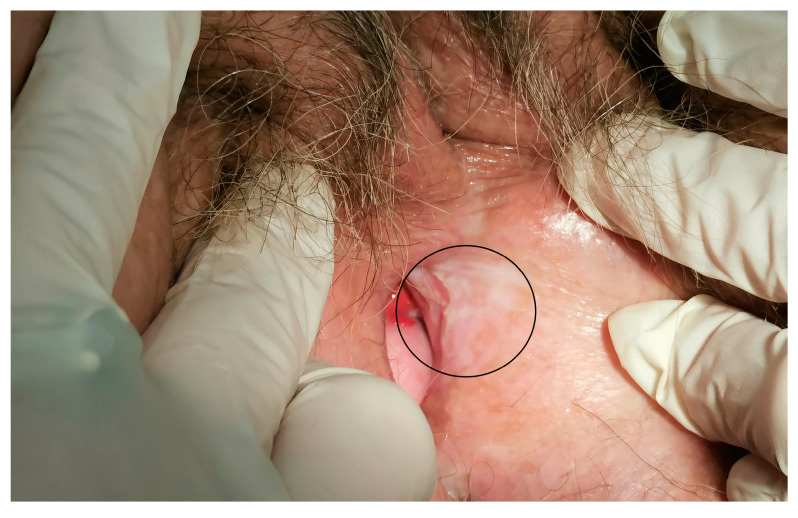
The area of electrochemotherapy treatment after 6 months (circled line).

The median maximum diameter of the lesions before electrochemotherapy was 12.5 (range 10–50) mm. Six patients were treated under general anesthesia and four patients were treated under regional (spinal) anesthesia. During the procedure, there was minimal blood loss from the application site. No intraoperative complications occurred. Postoperatively, during the hospital stay, patients did not experience local pain, fever, or nausea. Two to three weeks after electrochemotherapy, we observed mild pain at the application site. The pain was well-controlled with nonopioid analgesics per os. In two cases, in which larger skin ulcerations developed, patients required additional opioid analgesics per os for moderate pain. The skin ulcerations healed within 6 weeks by secondary intention. The cancer pain service and specialized wound care team were not involved or needed. In the case of urethral or periurethral involvement, a urinary catheter was placed in the bladder for a few days after electrochemotherapy to promote healing. The case of periurethral vulvar cancer recurrence is shown in Figure 1, and the postoperative result after six months in this specific area is shown in Figure 2.

No antibiotic therapy was administered. The median hospital stay was 2 days. All patients were admitted to the hospital one day before the treatment. The postoperative hospital stay was 24 h for most patients. Eight patients received a bleomycin dose of 15,000 IU/m^2^ and two patients received a bleomycin dose of 10,000 IU/m^2^. The lower dose of bleomycin was administered to two patients because of their older age and associated comorbidities. The median number of electric pulse applications was 6.5 (range 5–15). Each application consisted of 96 electric pulses at a frequency of 5 kHz when hexagonal geometry electrodes were used and 8 electrical pulses when finger electrodes were used. The highest applied current was less than 7 A in 6 patients and greater than 7 A in 4 patients. The most frequently used electrodes had a hexagonal geometry (nine patients); a finger electrode was used in one patient. In all patients, the complete area of the tumor was treated, demonstrating that with the selection of appropriate electrodes for a specific tumor size and location, the procedure is feasible. No electrochemotherapy-related or other serious adverse events occurred, demonstrating the safety of the treatment procedure. The detailed treatment data of the patients are shown in Table 3.

## 4. Discussion

To the best of our knowledge, this is the first prospective study of electrochemotherapy in the treatment of local vulvar cancer recurrence that is not used for palliative purposes. The main reason for treatment with electrochemotherapy was to avoid extensive and mutilating surgery. Our study proved that electrochemotherapy is a safe and feasible treatment method for local vulvar cancer recurrence.

Little is known about the safety and feasibility of electrochemotherapy treatment in gynecologic cancers. In the literature at present, there are only a few articles or clinical cases describing the use of electrochemotherapy in patients with vulvar or vaginal cancers for palliative purposes [15,16,17,18,19,20,21,22,23]. In these articles, detailed feasibility data are lacking. For vulvar cancer treatment with electrochemotherapy for palliative purposes, no treatment-related serious adverse events were reported in any of the studies. Some studies reported minor local adverse events, such as minimal blood loss, edema, erythema, hyperpigmentation, skin ulceration, and mild pain [24].

Electrochemotherapy is used, at present, in routine clinical practices for the treatment of cutaneous metastases of any histology and is included in the national and international guidelines for cutaneous metastases and primary skin cancer [11]. The safety and feasibility of electrochemotherapy were established based on the standard operating procedures. Electrochemotherapy is not associated with severe toxicity. Bleomycin is the most commonly used drug, due to its favorable toxicity profile. The toxicity of ECT is mostly associated with skin toxicity (hyperpigmentation, pruritus, hyperkeratosis, rash, erythema, etc.) occurring in up to 50% of patients. In less than 1% of cases, allergic reactions with fever, hypotension, or wheezing can occur shortly after treatment. In very rare cases, patients may experience lung toxicity (interstitial pneumonia that can evolve to lung fibrosis), which is commonly associated with IV bleomycin infusion and high cumulative dosages of bleomycin [14]. For cutaneous cancers, studies showed minimal serious adverse events, while the most common were mild, postprocedural nausea and dizziness [25]. According to a meta-analysis of superficial tumors, electrochemotherapy is associated with a 6% incidence of G3 toxicity, which is in line with other skin-directed therapies [14,26]. In other major series, relevant toxicity ranged from 0% to 18% and was manageable on an outpatient basis [14,27,28]. In the case of skin ulcerations, tissue healing occurred by secondary intention over 6–10 weeks, depending on tumor size and response [29]. In the treatment of ulcerated neoplastic wounds, the involvement of a specialized care team is advisable, together with a cancer pain service [14]. In general, during the inflammatory phase, treated skin should be covered with nonadherent, comfortable dressings, whereas ulcerated lesions are best managed by means of advanced wound dressings, such as alginates, charcoal, and silver. Tissue necrosis can be treated by enzymatic and/or surgical debridement to prevent superinfection and promote healing, according to the standard operating procedures for electrochemotherapy [11].

Extensive and mutilating surgery in women with recurrent vulvar cancer is associated with high morbidity and mortality and substantial treatment costs [30]. In cases of prior radiation, surgery is often the only treatment option for vulvar cancer recurrence. Pelvic exenteration is associated with complications in 57–82% of cases [30,31,32]. The most commonly reported complication was hemorrhage, followed by ileus of small bowel obstruction, wound complication, abdominal abscess, leakage of intestinal reconstruction, bowel perforation, respiratory failure, and acute renal injury. Sepsis, thromboembolism, and pneumonia occur in 6–8.4% of patients. Reoperation due to complications after pelvic exenteration is required in 12.4% of cases [32]. The reported mortality is 1.9–2.3%. In women with vulvar cancer recurrence, electrochemotherapy seems to be well-tolerated and has no serious adverse events [14]. As shown in our study, patients treated with electrochemotherapy had a short hospital stay compared with patients who underwent extensive and mutilating surgery [20]. The median hospital stay of our patients was two days; however, based on our safety results, the patient could be admitted and discharged from the hospital on the same day.

Women who undergo surgical treatment for vulvar cancer are at higher risk for lower quality of life in the form of psychological distress and sexual dysfunction and dissatisfaction with partner relationships [6]. Surgical procedures involving pelvic exenteration also lead to the formation of bowel and/or urinary stomas [33]. Exenterative surgery with short- and long-term adverse effects results in a significant impairment of quality of life. These inconveniences are particularly severe in younger patients. The formation of a bowel or urinary stoma can seriously affect social relationships [6].

Independent predictors of poor quality of life after extensive surgery include permanent colostomy and incontinent bladder, leading to financial burden, a negative attitude toward the disease, gastrointestinal symptoms, insomnia, depression, anxiety, and fatigue [6,31]. In addition, vulvar surgery negatively affects sexual life in 70% of cases; however, less radical procedures show better results and a reduced impact on quality of life. Whenever possible, partial vulvectomy is performed, attempting to preserve the clitoral hood [34]. The treatment of vulvar cancer has evolved toward conservative surgery in recent years. Electrochemotherapy could be considered such a conservative approach. After treatment with electrochemotherapy, the slow resolution of the treated tumor follows due to apoptotic cell death during cell division. In electrochemotherapy, cell death occurs due to the cytotoxicity of the drug, in our case bleomycin, and not due to electroporation, as in the case of irreversible electroporation [35,36,37].

To our knowledge, there is only one study reporting the effects of electrochemotherapy on the quality of life of patients with vulvar cancer for palliative purposes [38]. Patients with a single lesion, as in our study, reported a significant improvement in symptoms of bleeding, burning, and urination after electrochemotherapy, confirming the beneficial effects of electrochemotherapy on patients’ quality of life.

The safety profile of electrochemotherapy was favorable in our study. The treatment modality was acceptable and tolerable for all patients. No local or systemic adverse events were reported. Mild pain at the application site could be well-controlled with analgesics per os. In comparison, radical oncologic resection can be disfiguring and mutilating, whereas concurrent chemoradiotherapy can cause significant acute toxicity and potential late complications. An advantage of electrochemotherapy is that it can be performed easily and rapidly. To alleviate the pain associated with the application of the current pulses, local (spinal anesthesia) or general anesthesia can be used. In our experience, both anesthetic protocols were suitable to avoid the discomfort of muscle contractions at the time of current pulse delivery.

The feasibility of vulvar cancer treatment with electrochemotherapy was demonstrated by performing electrochemotherapy according to the standard operating procedure for electrochemotherapy [11]. In all patients, a complete tumor area was treated, which was achieved by the appropriate selection of electrodes based on the size and location of the tumor. In the treated area with safety margins, there were also the urethra and anal sphincter, whose functions were preserved by electrochemotherapy treatment. Electrochemotherapy showed good tissue preservation results and allowed urethral, vaginal, and anal sphincter resections to be avoided without postoperative complications. In addition, electrochemotherapy treatment provided better cosmetic results by reducing the extent of surgical resection. As a result, the negative impact on quality of life was reduced. In fact, several studies have shown that surgical aggressiveness is the main cause of deterioration in quality of life, and, therefore, individualized and more conservative surgical interventions or other treatment options should be an objective in the management of vulvar cancer [7,39,40,41].

The main limitation of our study is represented by the small number of treated patients. However, vulvar cancer recurrences are rare and difficult to treat. Therefore, even small prospective studies are important to verify the value of new treatment methods. The second limitation was the short-term follow-up, which was long enough to demonstrate the feasibility and safety of the treatment, which was our primary aim. Our study was a prospective study and is still ongoing. The third limitation was the lack of data on the effectiveness of electrochemotherapy treatment due to the short-term follow-up. However, based on the previous and latest data, the efficiency of electrochemotherapy treatment was over 90% [8].

The results of our study can be considered for future prospective studies to determine the best treatment option.

## 5. Conclusions

Our data show that electrochemotherapy is a feasible and safe technique for the treatment of vulvar cancer recurrence for nonpalliative purposes. Based on our results, electrochemotherapy might be used as a modern therapeutic tool for patients who would otherwise have to undergo surgery involving the mutilation of the external genitalia.

However, further studies are needed to analyze the oncological safety of this treatment.

## Figures and Tables

**Table 1:** Inclusion and exclusion criteria for electrochemotherapy in vulvar cancer.

***INCLUSION CRITERIA***
1. Local recurrence of vulvar cancer confirmed by histology
2. The largest diameter of tumor: 50 mm or less
3. Older than 18 years old
4. Life expectancy longer than 3 months
5. Performance status according to Karnofsky ≥ 70 or <2 according to the WHO scale
6. At least 2 weeks have passed since the last treatment
7. The patient must be able to understand the treatment process and possible side effects that may occur during the treatment
8. Signed informed consent form
9. The patient must be presented at a Multidisciplinary Tumor Board
***EXCLUSION CRITERIA***
1. Life-threatening infection and/or heart failure and/or liver failure and/or markedly impaired pulmonary function and/or other life-threatening systemic diseases
2. Regional or distant metastases
3. Younger than 18 years old
4. Major disturbances in the coagulation system (which do not respond to standard therapy—replacement of vitamin K or fresh frozen plasma)
5. Exposure to cumulative bleomycin doses higher than 400 mg
6. Impaired renal function (creatinine > 150 µmol/L)
7. Epilepsy
8. Pregnancy
9. Patients who are unable to understand the treatment process or refuse to be involved in the treatment process

**Table 2:** Baseline clinical characteristics of the study population.

Characteristics	Patients	Percentage
**Age**	**Years**	
Median	79.6	
Range	64–91	
**Histology**	10	
Squamous cell carcinoma		
**WHO performance status**	8	80%
0–1	2	20%
2	0	0%
3		
**Body mass index (BMI)**	**BMI**	
Median	24.96	
Range	16.3–32.86	
**Previous treatment**	6	60%
Surgery	4	40%
Surgery + Radiotherapy		

Legend: WHO: World Health Organization.

**Table 3:** Treatment data.

Characteristics	Patients	Percentage
**Tumor maximum diameter**	**Millimeters**	
Median	12.5	
Range	10–50	
**Anatomical site**		
Clitoris	1	10%
Paraurethral	3	30%
Labia minora	2	20%
Labia majora	2	20%
Perineum	2	20%
**Anesthesia**		
Local	4	40%
General	6	60%
**Cliniporator**		
IGEA Italy	10	100%
**Bleomycin dosage**		
15,000 IU/m^2^	8	80%
10,000 IU/m^2^	2	20%
**Type of electrodes used in electrochemotherapy**		
Hexagonal electrode	9	90%
Finger electrode	1	10%
**Area completely treated**		
Yes	10	100%
No	0	0%
**Number of applications (NoA)**	**(NoA)**	
Median	6.5	
Range	5–15	
**Highest current (A)**		
3–5	3	30%
5–7	3	30%
7–10	2	20%
Higher than 10	2	20%
**Toxicity (CTCAE grade)**	**Adverse Events**	
ECT-related	0	
Non-ECT-related within 24 h	0	
Non-ECT-related after 24 h	0	
**Duration of hospitalization**	**Days**	
Median	2	
Range	2–5	

Legend: IUs: international units; A: ampere; CTCAE: common terminology criteria for adverse events; ECT: electrochemotherapy.

## Data Availability

The data presented in this study are available on request from the corresponding author. The data are not publicly available due to the privacy of the patients.

## References

[1] Virarkar M., Vulasala S.S., Daoud T., Javadi S., Lall C., Bhosale P. (2022). Vulvar Cancer: 2021 Revised FIGO Staging System and the Role of Imaging. Cancers.

[2] Pedrão P.G., Guimarães Y.M., Godoy L.R., Possati-Resende J.C., Bovo A.C., Andrade C.E.M.C., Longatto-Filho A., Dos Reis R. (2022). Management of Early-Stage Vulvar Cancer. Cancers.

[3] Kang Y., Smith M., Barlow E., Coffey K., Hacker N., Canfell K. (2017). Vulvar Cancer in High-income Countries: Increasing Burden of Disease. Int. J. Cancer.

[4] Tan A., Bieber A.K., Stein J.A., Pomeranz M.K. (2019). Diagnosis and Management of Vulvar Cancer: A Review. J. Am. Acad. Dermatol..

[5] (2002). PDQ Adult Treatment Editorial Board Vulvar Cancer Treatment (PDQ®): Health Professional Version. PDQ Cancer Information Summaries.

[6] Malandrone F., Bevilacqua F., Merola M., Gallio N., Ostacoli L., Carletto S., Benedetto C. (2021). The Impact of Vulvar Cancer on Psychosocial and Sexual Functioning: A Literature Review. Cancers.

[7] Giannini A., D’Oria O., Chiofalo B., Bruno V., Baiocco E., Mancini E., Mancari R., Vincenzoni C., Cutillo G., Vizza E. (2022). The Giant Steps in Surgical Downsizing toward a Personalized Treatment of Vulvar Cancer. J. Obstet. Gynaecol. Res..

[8] Clover A.J.P., de Terlizzi F., Bertino G., Curatolo P., Odili J., Campana L.G., Kunte C., Muir T., Brizio M., Sersa G. (2020). Electrochemotherapy in the Treatment of Cutaneous Malignancy: Outcomes and Subgroup Analysis from the Cumulative Results from the Pan-European International Network for Sharing Practice in Electrochemotherapy Database for 2482 Lesions in 987 Patients (2008–2019). Eur. J. Cancer.

[9] Djokic M., Cemazar M., Bosnjak M., Dezman R., Badovinac D., Miklavcic D., Kos B., Stabuc M., Stabuc B., Jansa R. (2020). A Prospective Phase II Study Evaluating Intraoperative Electrochemotherapy of Hepatocellular Carcinoma. Cancers.

[10] Miklavčič D., Mali B., Kos B., Heller R., Serša G. (2014). Electrochemotherapy: From the Drawing Board into Medical Practice. BioMed Eng. OnLine.

[11] Gehl J., Sersa G., Matthiessen L.W., Muir T., Soden D., Occhini A., Quaglino P., Curatolo P., Campana L.G., Kunte C. (2018). Updated Standard Operating Procedures for Electrochemotherapy of Cutaneous Tumours and Skin Metastases. Acta Oncol..

[12] Michielin O., van Akkooi A.C.J., Ascierto P.A., Dummer R., Keilholz U., ESMO Guidelines Committee (2019). Electronic address: Clinicalguidelines@esmo.org Cutaneous Melanoma: ESMO Clinical Practice Guidelines for Diagnosis, Treatment and Follow-up. Ann. Oncol..

[13] Valpione S., Campana L.G., Pigozzo J., Chiarion-Sileni V. (2015). Consolidation Electrochemotherapy with Bleomycin in Metastatic Melanoma during Treatment with Dabrafenib. Radiol. Oncol..

[14] Campana L.G., Miklavčič D., Bertino G., Marconato R., Valpione S., Imarisio I., Dieci M.V., Granziera E., Cemazar M., Alaibac M. (2019). Electrochemotherapy of Superficial Tumors—Current Status:: Basic Principles, Operating Procedures, Shared Indications, and Emerging Applications. Semin. Oncol..

[15] Perrone A.M., Galuppi A., Cima S., Pozzati F., Arcelli A., Cortesi A., Procaccini M., Pellegrini A., Zamagni C., De Iaco P. (2013). Electrochemotherapy Can Be Used as Palliative Treatment in Patients with Repeated Loco-Regional Recurrence of Squamous Vulvar Cancer: A Preliminary Study. Gynecol. Oncol..

[16] Perrone A.M., Cima S., Pozzati F., Frakulli R., Cammelli S., Tesei M., Gasparre G., Galuppi A., Morganti A.G., De Iaco P. (2015). Palliative Electro-Chemotherapy in Elderly Patients with Vulvar Cancer: A Phase II Trial: Electro-Chemotherapy in Vulvar Cancer. J. Surg. Oncol..

[17] Perrone A.M., Galuppi A., Pirovano C., Borghese G., Covarelli P., De Terlizzi F., Ferioli M., Cara S., Morganti A.G., De Iaco P. (2019). Palliative Electrochemotherapy in Vulvar Carcinoma: Preliminary Results of the ELECHTRA (Electrochemotherapy Vulvar Cancer) Multicenter Study. Cancers.

[18] Tranoulis A., Georgiou D., Founta C., Mehra G., Sayasneh A., Nath R. (2020). Use of Electrochemotherapy in Women with Vulvar Cancer to Improve Quality-of-Life in the Palliative Setting: A Meta-Analysis. Int. J. Gynecol. Cancer.

[19] Corrado G., Cutillo G., Fragomeni S.M., Bruno V., Tagliaferri L., Mancini E., Certelli C., Paris I., Vizza E., Scambia G. (2020). Palliative Electrochemotherapy in Primary or Recurrent Vulvar Cancer. Int. J. Gynecol. Cancer.

[20] Vivod G., Kovacevic N., Čemažar M., Serša G., Jesenko T., Bošnjak M., Kranjc Brezar S., Merlo S. (2022). Electrochemotherapy as an Alternative Treatment Option to Pelvic Exenteration for Recurrent Vulvar Cancer of the Perineum Region. Technol. Cancer Res. Treat..

[21] Perrone A.M., Corrado G., Coada C.A., Garganese G., Fragomeni S.M., Tagliaferri L., Di Costanzo S., De Crescenzo E., Morganti A.G., Ferioli M. (2023). Electrochemotherapy with Intravenous Bleomycin for Heavily Pre-Treated Vulvar Cancer Patients. Int. J. Gynecol. Cancer.

[22] Vivod G., Jesenko T., Gasljevic G., Kovacevic N., Bosnjak M., Sersa G., Merlo S., Cemazar M. (2023). Treatment of Vulvar Cancer Recurrences with Electrochemotherapy—A Detailed Analysis of Possible Causes for Unsuccessful Treatment. Radiol. Oncol..

[23] Perrone A.M., Ferioli M., Galuppi A., Coe M., De Terlizzi F., Tesei M., Dondi G., De Palma A., Morganti A.G., De Iaco P. (2020). Palliative Treatment with Electrochemotherapy in Recurrent or Metastatic Vaginal Cancer. Int. J. Gynecol. Cancer.

[24] Certelli C., Garganese G., Fragomeni S., Tagliaferri L., Paris I., Gambacorta M., Scambia G., Corrado G. (2020). Electrochemotherapy in Vulvar Cancer: A Systematic Review. Ital. J. Gynaecol. Obstet..

[25] Morley J., Grocott P., Purssell E., Murrells T. (2019). Electrochemotherapy for the Palliative Management of Cutaneous Metastases: A Systematic Review and Meta-Analysis. Eur. J. Surg. Oncol..

[26] Spratt D.E., Gordon Spratt E.A., Wu S., DeRosa A., Lee N.Y., Lacouture M.E., Barker C.A. (2014). Efficacy of Skin-Directed Therapy for Cutaneous Metastases From Advanced Cancer: A Meta-Analysis. J. Clin. Oncol..

[27] Kunte C., Letulé V., Gehl J., Dahlstroem K., Curatolo P., Rotunno R., Muir T., Occhini A., Bertino G., Powell B. (2017). Electrochemotherapy in the Treatment of Metastatic Malignant Melanoma: A Prospective Cohort Study by InspECT. Br. J. Dermatol..

[28] Matthiessen L.W., Johannesen H.H., Hendel H.W., Moss T., Kamby C., Gehl J. (2012). Electrochemotherapy for Large Cutaneous Recurrence of Breast Cancer: A Phase II Clinical Trial. Acta Oncol..

[29] Heller R., Jaroszeski M.J., Reintgen D.S., Puleo C.A., DeConti R.C., Gilbert R.A., Glass L.F. (1998). Treatment of Cutaneous and Subcutaneous Tumors with Electrochemotherapy Using Intralesional Bleomycin. Cancer.

[30] Matsuo K., Mandelbaum R.S., Adams C.L., Roman L.D., Wright J.D. (2019). Performance and Outcome of Pelvic Exenteration for Gynecologic Malignancies: A Population-Based Study. Gynecol. Oncol..

[31] Stanca M., Căpîlna D.M., Căpîlna M.E. (2022). Long-Term Survival, Prognostic Factors, and Quality of Life of Patients Undergoing Pelvic Exenteration for Cervical Cancer. Cancers.

[32] Tortorella L., Casarin J., Mara K.C., Weaver A.L., Multinu F., Glaser G.E., Cliby W.A., Scambia G., Mariani A., Kumar A. (2019). Prediction of Short-Term Surgical Complications in Women Undergoing Pelvic Exenteration for Gynecological Malignancies. Gynecol. Oncol..

[33] de Gregorio N., de Gregorio A., Ebner F., Friedl T.W.P., Huober J., Hefty R., Wittau M., Janni W., Widschwendter P. (2019). Pelvic Exenteration as Ultimate Ratio for Gynecologic Cancers: Single-Center Analyses of 37 Cases. Arch. Gynecol. Obstet..

[34] Zeitoun J., Calvary M., Bonneau C., Rouzier R. (2022). Impact of Vulvar Cancer Surgery on Quality of Sex Life: A Review of Literature. J. Low Genit. Tract. Dis..

[35] Geboers B., Scheffer H.J., Graybill P.M., Ruarus A.H., Nieuwenhuizen S., Puijk R.S., van den Tol P.M., Davalos R.V., Rubinsky B., de Gruijl T.D. (2020). High-Voltage Electrical Pulses in Oncology: Irreversible Electroporation, Electrochemotherapy, Gene Electrotransfer, Electrofusion, and Electroimmunotherapy. Radiology.

[36] Batista Napotnik T., Polajžer T., Miklavčič D. (2021). Cell Death Due to Electroporation—A Review. Bioelectrochemistry.

[37] Wijayanta A.T., Kurata K. (2023). Comprehensive Review on Thermal Aspects of Nonthermal Irreversible Electroporation. Heat Transf..

[38] Perrone A.M., Ferioli M., Argnani L., De Terlizzi F., Pirovano C., Covarelli P., Dondi G., Tesei M., De Crescenzo E., Ravegnini G. (2021). Quality of Life with Vulvar Carcinoma Treated with Palliative Electrochemotherapy: The ELECHTRA (ELEctroCHemoTherapy VulvaR CAncer) Study. Cancers.

[39] Günther V., Malchow B., Schubert M., Andresen L., Jochens A., Jonat W., Mundhenke C., Alkatout I. (2014). Impact of Radical Operative Treatment on the Quality of Life in Women with Vulvar Cancer--a Retrospective Study. Eur. J. Surg. Oncol..

[40] Rogers L.J. (2021). Management of Advanced Squamous Cell Carcinoma of the Vulva. Cancers.

[41] O’Donnell R.L., Verleye L., Ratnavelu N., Galaal K., Fisher A., Naik R. (2017). Locally Advanced Vulva Cancer: A Single Centre Review of Anovulvectomy and a Systematic Review of Surgical, Chemotherapy and Radiotherapy Alternatives. Is an International Collaborative RCT Destined for the “Too Difficult to Do” Box?. Gynecol. Oncol..

